# Supplementary material for: Merqury: reference-free quality, completeness, and phasing assessment for genome assemblies
Source: Genome Biol. 2020 Sep 14;21:245. doi: 10.1186/s13059-020-02134-9 (PMC7488777; doi:10.1186/s13059-020-02134-9)
Supplement: Supplementary file 1 — Additional file 1. Review history. [file 13059_2020_2134_MOESM1_ESM.docx]

Review History

**First round of review**

**Reviewer 1**

**Comments to author:**

When a new genome is sequenced, it needs to be validated. As the genome is new there is no reference sequence available. Traditional ways of assessing the quality of a new genome rely on mapping reads back to the genome or evaluating the presence of common genes in the newly assembled genome. Validation of genome assemblies is also needed when new assemblers are developed. For a long time, validation in this context has relied on using well curated reference sequences. As long read assemblies are now becoming more complete than the available references (e.g. telomer-to-telomer assemblies and haplotype resolved assemblies), reference free validation tools are becoming more important also in this application.

In this paper the authors propose a new reference free tool for the validation of genome assemblies. The approach they take is to compare the k-mer spectrum of the assembly to that of a highly accurate short read data set of the same individual. This approach has been previously used in the KAT tool. The KAT tool focused on evaluating consensus assemblies. Here this approach is extended to haplotype resolved assemblies. Additionally the authors show how the base level quality of an assembly (QV) can be assessed from the k-mer spectra.

Major:

1) On lines 23-25 the author criticise reference based validation by saying that it reports true variants as potential mis-assemblies. What is meant by mis-assemblies here? Commonly mis-assemblies are defined as repeat collapses and expansion, and sequence rearrangements and inversions. It is true that if the sequenced genome has this kind of true variants, reference based methods will report them as potential mis-assemblies. However, k-mer based methods such as the one introduced in this paper, cannot detect sequence rearrangements and inversions at all because they do not change the k-mer spectra of the sequence. To be fair, this limitation of the approach taken in this paper should be mentioned.

2) The copy number spectrum (spectra-cn) plots discussed at length on pages 6-10 seem to be exactly the same kind of plots produced by KAT. While these plots are informative, they are not novel to the new tool. Here it would also be interesting to compare against KAT. The resulting plot should be the same but how does the runtime and memory usage of Merqury compare to KAT?

3) On page 18 the authors state that if resources are scarce, Mash could be used instead of Meryl. In the light of this I find the results in Table 3 confusing. I understand that Meryl based analysis is split into three phases (count, union-sum and QV(Meryl) in Table 3. Is this so? How does QV(Mash) column fit into this? In order to run QV analysis with Mash does one need to first do the two first phases Count and Union-sum? Also the runtime of QV(Mash) seems really high. Please clarify.

4) The paragraph on lines 215-224 is not self contained but requires the reader to consult the legend of Figure 4. Please expand the paragraph by explaining the corresponding plot in more detail.

Minor:

Line 63: Is the reference for Arabidopsis thaliana F1 hybrid correct?

Line 421: size if k -> size of k

Line 456: this is a rare -> this is rare

**Reviewer 2**

**Comments to author:**

The manuscript describes Merqury, a tool for genome assembly validation, and quality estimation based on k-mers. While there have been tools published for this before, Merqury includes novel features like consensus quality, k-mer completeness and perhaps as the most interesting part completeness/correctness statistics for haplotype assemblies. The manuscript is well written, and explains problems inherent to genome assembly along with the metrices to analyze them in a very accessible way.

Table 1 shows that Merqury extends the k-mer set operation tool KAT with features for genome quality estimation, as KAT’s features are not listed under Assembly statistics. However KAT does already include statistics for genome assembly evoluation and is not only a k-mer set operation tool.

While the manuscript describes applications of Merqury, it is not clear what the algorithms behind the k-mer set operations are and how their performance is. For example, do the set operation involve false positives or false negatives?

"Mercury's methods are extensible to polyploid genomes" Would it be possible to give a brief description what the extension to polyploids would involve?

Minor:

L123-125 and 182-183: How much would genome polishing affect assembly quality and its assessment?

L134-135: kbp is not a fraction.

L200-201: “A human genome, in comparison, has many fewer hap-mers,….”. Relatively? Humans still should have more in absolute numbers.

Fig. 4b. No units for “Total, Max, NG50”

**Authors Response**

**Point-by-point responses to the reviewers’ comments:**

Reviewer #1 comments

Reviewer #1: When a new genome is sequenced, it needs to be validated. As the genome is new there is no reference sequence available. Traditional ways of assessing the quality of a new genome rely on mapping reads back to the genome or evaluating the presence of common genes in the newly assembled genome. Validation of genome assemblies is also needed when new assemblers are developed. For a long time, validation in this context has relied on using well curated reference sequences. As long read assemblies are now becoming more complete than the available references (e.g. telomer-to-telomer assemblies and haplotype resolved assemblies), reference free validation tools are becoming more important also in this application.

In this paper the authors propose a new reference free tool for the validation of genome assemblies. The approach they take is to compare the k-mer spectrum of the assembly to that of a highly accurate short read data set of the same individual. This approach has been previously used in the KAT tool. The KAT tool focused on evaluating consensus assemblies. Here this approach is extended to haplotype resolved assemblies. Additionally the authors show how the base level quality of an assembly (QV) can be assessed from the k-mer spectra.

Major:

1) On lines 23-25 the author criticise reference based validation by saying that it reports true variants as potential mis-assemblies. What is meant by mis-assemblies here? Commonly mis-assemblies are defined as repeat collapses and expansion, and sequence rearrangements and inversions. It is true that if the sequenced genome has this kind of true variants, reference based methods will report them as potential mis-assemblies. However, k-mer based methods such as the one introduced in this paper, cannot detect sequence rearrangements and inversions at all because they do not change the k-mer spectra of the sequence. To be fair, this limitation of the approach taken in this paper should be mentioned.

*We agree that k-mer-based methods are limited in their ability to precisely identify some structural mis-assemblies using short reads, and have expanded the Introduction and Discussion to include limitations of Merqury.*

*Added to the Introduction:*

*A reference-based approach has the advantage of detecting structural mis-assemblies that gene or k-mer based methods may miss, but reliance on a reference alignment can result in true variants being reported as potential mis-assemblies. A recent extension to the QUAST toolkit combines evidence from both k-mers and long reads to better identify certain classes of structural mis-assembly19.*

*Added to the Discussion:*

*Currently, Merqury does not explicitly validate the structural accuracy of an assembly, which is another important factor of assembly quality32. Although certain mis-assemblies, such as the systematic collapse of repeats, would be visible from the spectra-cn plots, other types of structural mis-assemblies, such as inversions, could go unnoticed. This is one drawback of Merqury’s k-mer-centric design. However, we have observed that regions of mis-assembly often correlate with a decrease in consensus quality, similar to the haplotype switch example shown in Figure 5. This information can be used to draw attention to unreliable regions of the assembly that can be later validated using orthogonal approaches.*

2) The copy number spectrum (spectra-cn) plots discussed at length on pages 6-10 seem to be exactly the same kind of plots produced by KAT. While these plots are informative, they are not novel to the new tool. Here it would also be interesting to compare against KAT. The resulting plot should be the same but how does the runtime and memory usage of Merqury compare to KAT?

*We agree providing a breakdown of the runtime and memory usage comparison between Merqury and KAT would be useful and have added a comparison of KAT and Merqury runtimes to the Results section:*

*We also compared CPU time and maximum memory used by Merqury and KAT comp to generate the same spectra-cn plots for the TrioCanu assembly of NA12878. Here, we compared Merqury and KAT comp for a single haploid assembly (maternal) and for the diploid assembly, which includes the maternal, paternal, and combined haplotype assemblies (Table 4). When run on the haploid assembly, Merqury was able to generate a spectra-cn plot and all other evaluation metrics using half the memory (21.2 GB vs 44.1 GB) and comparable runtime to KAT, which generates only a spectra-cn plot (22.5 CPU h vs 22.4 CPU h). Mercury runtime includes the construction of a k-mer count database of the reads, which must only be generated once. Thus, when using a pre-built database, the run time requirement drops by more than half (8.7 CPU h per assembly). In comparison, KAT must recount k-mers in the reads for each spectra-cn plot, and as a result, the runtime of KAT triples to 63.0 CPU h when evaluating the diploid assembly. Because Merqury utilizes efficient set operations on k-mer databases, k-mer counting of each haploid assembly needs only be performed once, internally computing k-mers of the combined assembly. The diploid assembly can be analyzed in 32.5 CPU h, or 19.7 CPU h with a pre-built database, including the generation of Merqury’s additional quality and phasing metrics.*

*We note that the copy-number spectrum (spectra-cn) is the only overlapping functionality provided by KAT. Merqury introduces new features such as spectra-asm, spectra-hap, quality, and k-mer completeness measurements, and provides more flexibility in plotting the results of these analyses. We also provide a thorough explanation of how to interpret these k-mer-based results in real assembly contexts.*

3) On page 18 the authors state that if resources are scarce, Mash could be used instead of Meryl. In the light of this I find the results in Table 3 confusing. I understand that Meryl based analysis is split into three phases (count, union-sum and QV(Meryl) in Table 3. Is this so? How does QV(Mash) column fit into this? In order to run QV analysis with Mash does one need to first do the two first phases Count and Union-sum? Also the runtime of QV(Mash) seems really high. Please clarify.

*Mash replaces all three steps of the Meryl-based QV estimate. Please refer to the Mash Screen paper for details on the method, but to summarize, Mash retains only a subset of the assembly k-mers in a dimensionality-reduced “sketch” data structure. To estimate the QV, all k-mers from the reads are then streamed against this sketch to determine which of the sketch’s k-mers are also found in the reads. The increased runtime is the result of streaming all reads from disk and looking them up in a hash table of sketch values, versus constructing and comparing sorted k-mer databases, which requires more memory and disk but less time. We have modified the table header so it clearly distinguishes what is run under Merqury and modified the caption:*

*All statistics are for the diploid (maternal, paternal, and combined) assembly of the human genome NA12878. Merqury QV estimates are generated from the full k-mer databases and use exact k-mer counting, whereas Mash QV estimates are generated by streaming all reads against a MinHash sketch of the assembly. Merqury’s Count and Union-sum steps count all k-mers in the reads, while the QV estimation counts k-mers in the assembly and compares these to the read counts. Mash’s QV estimation creates a k-mer sketch for the assembly and streams all reads against the sketch. Results are totalled over three QV operations (maternal, paternal, and combined). Runtimes were measured on Intel(R) Xeon(R) Gold 6140 CPU, with 2.30GHz. Storage requirements represent gzipped FASTQ files for counting and QV (Mash), and a binary database for QV (Meryl). h, hours; m, minutes; s, seconds; G, Giga bytes.*

4) The paragraph on lines 215-224 is not self contained but requires the reader to consult the legend of Figure 4. Please expand the paragraph by explaining the corresponding plot in more detail.

*We have revised this paragraph as follows:*

*Visualizing hap-mer presence in each haplotype assembly is also useful to detect overall phase consistency. When counting Col- and Cvi-specific k-mers in contigs of the TrioCanu assembly (Fig. 4c), each contig was successfully separated by haplotype as expected. That is, the Col markers were observed in the Col haplotype assembly, with almost no contaminating Cvi markers, and vice versa. The count of the found k-mers can be used as the x and y axis when plotting each contig as a blob. The blob is colored by the origin of the assembly, with the size being relative to the contig size. A haplotype resolved assembly with almost no haplotype switches is expected to have all blobs close to either one of the axes, with the colors matching the corresponding haplotype of the assembly. The FALCON-Unzip alternate contigs maintain haplotype consistency (Fig. 4d), whereas the primary pseudo-haplotype contigs contain a mixture of k-mers from both haplotypes. The Canu assembly mixes haplotypes in all but the smallest contigs (Fig. 4e), and would require partitioning into primary and alternate contig sets using a post-processing tool such as purge_dups35.*

Minor:

Line 63: Is the reference for Arabidopsis thaliana F1 hybrid correct?

*We thank the reviewer for noticing this mistake. The reference has been corrected.*

Line 421: size if k -> size of k

*Fixed.*

Line 456: this is a rare -> this is rare

*Fixed.*

Reviewer #2 comments

Reviewer #2: The manuscript describes Merqury, a tool for genome assembly validation, and quality estimation based on k-mers. While there have been tools published for this before, Merqury includes novel features like consensus quality, k-mer completeness and perhaps as the most interesting part completeness/correctness statistics for haplotype assemblies. The manuscript is well written, and explains problems inherent to genome assembly along with the metrices to analyze them in a very accessible way.

*Thank you!*

Table 1 shows that Merqury extends the k-mer set operation tool KAT with features for genome quality estimation, as KAT’s features are not listed under Assembly statistics. However KAT does already include statistics for genome assembly evaluation and is not only a k-mer set operation tool.

*We agree that Table 1 could be more precise and have revised it accordingly.*

While the manuscript describes applications of Merqury, it is not clear what the algorithms behind the k-mer set operations are and how their performance is. For example, do the set operation involve false positives or false negatives?

*All k-mer operations performed by Meryl use exact algorithms (i.e. no false positives or negatives). The performance of Meryl is on par with other exact k-mer counters and the algorithms used are fairly standard. In response to Reviewer #1, we have now included a runtime comparison to KAT (Table 4), which demonstrates that Merqury is more than twice as fast. The subsection “Counting k-mers with Meryl” describes the method, which we have edited to clarify that all algorithms used are exact.*

"Mercury's methods are extensible to polyploid genomes" Would it be possible to give a brief description what the extension to polyploids would involve?

*Most of the operations would work without any extensions. However, we currently identify haplotype-specific k-mers (“hap-mers”) by assuming two parental genomes. This works well because diploid genomes inherit a single haplotype from each parent. In a polyploid species such as allohexaploid wheat, with six sets of chromosomes originating from three ancestral diploid species, identifying hap-mers would be complicated by the presence of six haplotypes rather than two. This would result in multiple heterozygous peaks, rather than one in the diploid case, but Merqury could still identify k-mers that were inherited exclusively from one parent. We have added a short clarification to the discussion:*

*Merqury is also extensible to polyploid genomes for most of the evaluations. However, for the phasing measurements, Merqury currently reports at most two parental (or ancestral) haplotypes. In the case of a polyploid genome, Merqury’s reported phase blocks would reflect inheritance from a single parent, but not necessarily the correct phasing of homologous chromosomes (e.g. two homologous chromosomes inherited from the same parent would be indistinguishable). To accurately measure the phasing of homologous chromosomes in a polyploid genome would require a different method for identifying hap-mers that does not rely solely on parental inheritance. To support alternative k-mer classification methods (e.g. Hi-C, Strand-seq, linked reads), Merqury is designed to receive any pre-computed hap-mer set as input.*

Minor:

L123-125 and 182-183: How much would genome polishing affect assembly quality and its assessment?

*Depending on how it is performed, genome polishing can both help and hurt the quality of an assembly. The assessment is independent and will measure which it is. Anecdotally, we have successfully used Merqury to identify and correct a deficiency of the PacBio/Arrow-based polishing pipeline used by the Vertebrate Genomes Project.*

L134-135: kbp is not a fraction.

*We have removed this parenthetical from the text.*

L200-201: “A human genome, in comparison, has many fewer hap-mers,….”. Relatively? Humans still should have more in absolute numbers.

*Correct, the number of hap-mers in this sentence was meant to be relative to all k-mers, not the absolute numbers. We modified the sentence accordingly:*

*“A human genome, in comparison, has relatively fewer hap-mers compared to all k-mers in the 1- and 2-copy peaks, with most of the k-mers shared between both haplotypes (Fig. 3d).”*

Fig. 4b. No units for “Total, Max, NG50”

*We thank the reviewer for noting the missing units (Mbp). We have added it to Fig. 4b.*

**Second round of review**

**Reviewer 1**

All my concerns have been answered.
